# Racial and Ethnic Disparities in Human Papillomavirus Vaccination Among US-Born and Foreign-Born Adults Aged 18 to 26 Years in the United States

**DOI:** 10.3390/vaccines13020098

**Published:** 2025-01-21

**Authors:** Itunu Sokale, Jane Montealegre, Ann O. Amuta, Abiodun Oluyomi, Aaron P. Thrift

**Affiliations:** 1 Department of Medicine, Section of Epidemiology and Population Sciences, Baylor College of Medicine, Houston, TX 77030, USAaaron.thrift@bcm.edu (A.P.T.); 2 Dan L. Duncan Comprehensive Cancer Center, Baylor College of Medicine, Houston, TX 77030, USA; 3 Department of Behavioral Science, The University of Texas MD Anderson Cancer Center, Houston, TX 77030, USA; 4 Department of Kinesiology Public Health Program, The University of Texas at Arlington, Arlington, TX 76019, USA

**Keywords:** human papillomavirus, HPV vaccination, vaccine inequalities, young adults, foreign-born populations, racial and ethnic minority populations

## Abstract

Background/Objectives: Human papillomavirus (HPV) is linked to multiple cancers that can be prevented through vaccination. While the optimal age for vaccination is in childhood and adolescence, vaccination recommendations include adults through age 26 who missed childhood/adolescent vaccination. There are limited data about disparities among adults eligible for catch-up HPV vaccination. We conducted a comprehensive examination of HPV vaccination among US young adults, disaggregating the group by race/ethnicity and nativity status to identify subgroups that may require additional interventions. Methods: We analyzed 2019 and 2022 data of individuals aged 18–26 years from the National Health Interview Survey. Generalized linear models using Poisson regression with log link were used to examine the receipt of 1+ dose of HPV vaccine, race/ethnicity, and nativity (i.e., US- versus foreign-born) status. Results: The overall receipt of 1+ doses of HPV vaccine was 47.5%. The vaccination rate among the US-born group was 49.7% versus 31.9% among the foreign-born group with an adjusted prevalence ratio (APR) of 0.72; (95% CI, 0.62–0.82). Foreign-born non-Hispanic (NH) Black individuals (APR 0.31; 95% CI, 0.13–0.70) were less likely to be vaccinated against HPV than foreign-born NH White individuals, while US-born NH Asians (APR 1.27; 95% CI, 1.09–1.48) had a higher prevalence of the vaccination than the US-born NH White group. Additionally, foreign-born NH Asian (APR 0.60; 95% CI, 0.46–0.77), NH Black (APR 0.27; 95% CI, 0.12–0.61), and Hispanic (APR 0.76; 95% CI, 0.60–0.97) populations were less likely to be vaccinated than their respective US-born counterparts. **Conclusion**: Profound HPV vaccination inequalities exist among US young adults with particularly low vaccine coverage among racially and ethnically minoritized immigrant populations.

## 1. Introduction

Human papillomavirus (HPV) is the leading cause of sexually transmitted infection in the United States. The lifetime risks of acquiring HPV infection exceed 80% for women and 90% for men [1], and an estimated 13 million people are newly infected annually [2]. Most HPV infections are asymptomatic, and regression usually occurs within two years, probably due to immunologic control, although some infections persist [3]. Persistent infection with high-risk HPV types can lead to clinical diseases, including anogenital warts, respiratory papillomatosis (a disease that can lead to benign tumor in the respiratory tract) [4], precancerous lesions, and cancers [3]. About 14 high-risk (oncogenic) HPV subtypes have been identified and classified as carcinogenic (subtypes 16 and 18), probably carcinogenic (subtypes 31 and 33), and possibly carcinogenic (some subtypes excluding 16, 18, 31, and 33) [3]. Persistent infections with high-risk HPV subtypes, particularly subtype 16 and 18, have been associated with multiple cancers, including cancers of the cervix, vagina, vulva, penis, anus, and oropharynx [3]. HPV causes approximately 5% of all cancers worldwide, and over 37,000 cancers are attributable to HPV each year in the US [5]. Non-Hispanic (NH) Black and Hispanic individuals bear a disproportionately higher burden of HPV-related cancers than NH White individuals [6].

Advancements in scientific evidence linking HPV to cancers have led to landmark discoveries, including HPV vaccination for the primary prevention of HPV-related cancers [7]. With more than 15 years of research and over 135 million HPV vaccine doses administered [8], the vaccine is safe, efficacious, and cost effective for preventing over 90% of new HPV infections, HPV-related precancerous lesions and cancers [9,10,11]. Past studies indicate that the HPV vaccine offers long-lasting protection against HPV infection and therefore disease caused by HPV infection [9]. Results of studies comparing the pre- and post-HPV vaccine era showed significant decreases in cervical cancer incidence rates among young females following the introduction of the vaccine [12,13]. A study reported that cervical cancer incidence rates were 29% lower in 2011–2014 (post-HPV-vaccine era, 6.0 per 1,000,000 people) compared to 2003–2006 (pre-HPV-vaccine era, 8.4 per 1,000,000 people) among women aged 15 through 24 years [12].

The HPV vaccine is most cost effective when given prior to sexual debut [14]. Thus, the Advisory Committee on Immunization Practices (ACIP) recommends HPV vaccination for children and adolescents at age 11 or 12 years and can be started at age 9 years [15]. Additionally, catch-up vaccination is recommended for unvaccinated persons through age 26 years and is effective in preventing new HPV infections and HPV-associated diseases in this age group as well [11,16]. Catch-up vaccination has been available in the US for females through age 26 years since the vaccine was first introduced in 2006 and was subsequently broadened to include males of the same age range in 2009 [17]. For adults ages 27–45 years, HPV vaccination can be given based on shared decision making between the clinician and the patient based on individual risk prediction and potential benefit [10,15]. A two-dose series over 6 to 12 months (0, 6–12 months) is recommended for most individuals who initiate HPV vaccination at ages 9–14 years, while a three-dose series (0, 1–2, 6 months) is administered to persons who commence the vaccination at 15–45 years and immunocompromised persons [18]. Most health insurance plans cover the HPV vaccine, and there are programs providing free vaccines to uninsured and underinsured children and teenagers in the US [19].

Overall, HPV vaccine uptake among children and adolescents in the US is suboptimal with 56.6% of boys and 60.7% of girls up to date on HPV vaccination [20]. Significant disparities exist across sociodemographic groups [20,21]. Prior studies have found a reduced likelihood of HPV vaccination among adolescents from racial and ethnic minority groups [22,23] due to multiple reasons. Prior studies have suggested that NH Black and Hispanic parents of age-eligible children and adolescents lacked HPV vaccination knowledge, were less likely to receive provider recommendations, thought HPV vaccination was not necessary, and had higher healthcare system mistrust [24,25]. Less is known about disparities among adults eligible for catch-up HPV vaccination.

In addition to racial/ethnic disparities [26], there are known inequalities in access to preventive care services between U.S.- and foreign-born individuals [27]. Foreign-born people account for 14.4% of the US population and are the fastest growing population in the US [28]. There were over 47.8 million foreign-born individuals in the US in 2023 compared to 9.6 million in 1970 [28]. Priors studies have indicated that HPV infection is prevalent among foreign-born individuals in the US [29], and they often present with late-stage HPV-related cancers at diagnosis [30], primarily due to lack of HPV prevention knowledge and access, including HPV vaccination. Nonetheless, nativity-related HPV vaccination disparities are often masked in analyses grouping U.S. and foreign-born individuals of a given race/ethnicity within a homogenous racial/ethnic category [31]. This may hinder the determination of subgroups needing additional interventions and the development and implementation of strategic and effective interventions to reduce HPV vaccination disparities. Thus, the current study examined HPV vaccination rates among adults aged 18–26 years by race and ethnicity disaggregated by nativity status to identify subgroups that may be targeted for aggressive HPV vaccination interventions to reduce HPV-related diseases disparities.

## 2. Materials and Methods

### Study Design, Data Source, and Population

In this population-based cross-sectional study, we analyzed data from the Integrated Public Use Microdata Series (IPUMS) Health Surveys, which is a harmonized version of the annual National Health Interview Survey (NHIS) [32]. The NHIS is a nationally representative cross-sectional survey of the United States (US) civilian noninstitutionalized population. The NHIS is primarily a phone survey that collects data on a broad range of health issues, healthcare utilization, and demographic and socioeconomic characteristics of survey respondents. NHIS data are publicly available and de-identified, and thus this study was exempt from review by the institutional review board as nonhuman subject research. The analysis data for the present study were derived from the 2019 and 2022 datasets. In 2019, 31,997 sample adults were interviewed (response rate: 61.1%), while in 2022, 27,651 sample adults were surveyed (response rate: 47.7%). Additional details on the sampling method, response rates, data quality, and weighting of NHIS data have been published elsewhere [32]. Data collected prior to 2019 were excluded due to the 2019 NHIS questionnaire redesigning for quality improvement, which may affect the comparability and interpretation of data collected before and after 2019. Additionally, we excluded 2020 and 2021 data due to COVID-19 pandemic disruptions of data collection [33]. To assess HPV vaccination rates among US young adults aged 18 through 26 years, we pooled and analyzed data from the 2019 and 2022 NHIS datasets through IPUMS (N = 5424). The survey weight for the pooled dataset was calculated and provided by IPUMS. We excluded respondents without HPV vaccination initiation information (*n* = 555), nativity status (*n* = 111), and selected variables, e.g., sex, health insurance (*n* = 34). The final sample included 4724 respondents aged 18–26 years. We followed the STROBE (Strengthening the Reporting of Observational Studies in Epidemiology) reporting guideline.

Outcome Variable: Self-reported receipt of at least 1 dose of the HPV vaccine was the outcome of interest. This was assessed in the NHIS by asking participants “Have you ever received an HPV vaccine?” Participants who reported ‘Yes’ to this question were considered HPV vaccinated, and those who reported ‘No’ were considered non-HPV vaccinated. Nearly 90% of respondents responded to this question.

Exposure Variables: Race/ethnicity and nativity status were the exposure variables of interest. All variables were self-reported. We categorized participants into five mutually exclusive racial/ethnic groups: Hispanic (any race), NH White, NH Black, NH Asian, and NH Other. Nativity status was based on respondents’ reports and classified as US-born and foreign-born. Respondents were considered foreign-born if they reported that they were not born in the United States or any of its territories.

Covariates: Potential confounders were self-reported and selected a priori [34], and they included sex (male, female), poverty–income ratio (<100% of poverty level, 100–199% of poverty level, ≥200% of poverty level), health insurance (yes, no), and US census region of residence (northeast, north central/Midwest, south, west).

Statistical analysis: All data analyses included sampling weight for the study years and accounted for complex sampling design. Weighted descriptive statistics were used to summarize HPV vaccination by demographic characteristics, including Person χ2 test. Unadjusted and adjusted generalized linear models using Poisson regression with log link were used to examine the associations between race/ethnicity and nativity status and receipt of at least 1 dose of the HPV vaccine. Analyses were adjusted for sex, poverty level, health insurance, and US census region of residence [34]. The statistical significance level was defined as 2-sided *p* < 0.05. Statistical analyses were performed using Stata Software Version 18.0 (StataCorp LLC, College Station, TX, USA).

## 3. Results

### 3.1. Description of Study Participants

Table 1 presents the weighted estimates of the characteristics of the study respondents (N = 4724) included in the analysis. Most were insured (84.7%), US-born (84.7%), NH White (54.0%), and were at ≥200% poverty level (63.7%). Overall, 47.5% of individuals received at least 1 dose of the HPV vaccine. Those who reported being vaccinated against HPV differed significantly from those who were not vaccinated by sex, race/ethnicity, poverty–income ratio, health insurance, nativity status, and geography.

**Figure 1:**
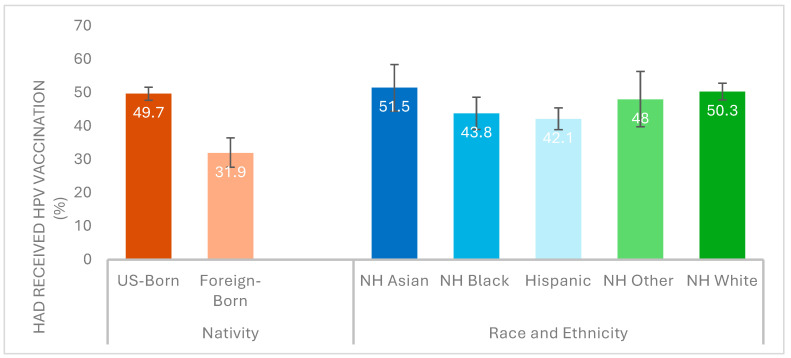
Weighted HPV vaccination rates among US young adults aged 18–26 years, by nativity and race/ethnicity. Abbreviations: HPV, human papillomavirus; NH, non-Hispanic. Race/ethnicity groups are mutually exclusive, and Hispanic/Latinos could be of any race. Other includes American Indian/Alaska Native, Asian, Native Hawaiian/Pacific Islander, and multiple races. Respondents were considered foreign-born if they reported that they were not born in the United States or any of its territories.

Across race/ethnicity, vaccination rate was highest in the NH Asian group (51.5%), which was followed by the NH White (50.3%), NH Other race (48%), NH Black (43.8%) and Hispanic (42.1%) groups (Figure 1). The vaccination rate in the US-born group was 49.7% versus 31.9% in the foreign-born group. Females had a higher vaccination rate (57%) than males (37.8%).

**Figure 2:**
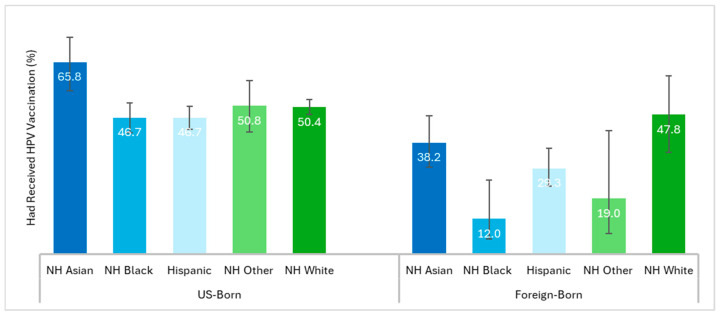
Weighted HPV vaccination rates among racial–ethnic groups of US young adults aged 18–26 years stratified by nativity. Abbreviations: HPV, human papillomavirus; NH, non-Hispanic; Race/ethnicity groups are mutually exclusive, and Hispanic/Latinos could be of any race; Other includes American Indian/Alaska Native; Asian, Native Hawaiian/Pacific Islander, and multiple races. Respondents were considered foreign-born if they reported that they were not born in the United States or any of its territories.

Disaggregated statistics showed heterogeneity in vaccination rates within each race/ethnicity category depending on nativity status (Figure 2). For instance, the vaccination rate was 65.8% for the US-born NH Asian group vs. 38.2% in the foreign-born NH Asian group, 46.7% for the US-born NH Black group vs. 12.0% in the foreign-born NH Black group, and 46.7% for the US-born Hispanic group vs. 29.3% in the foreign-born Hispanic group.

### 3.2. Unadjusted and Adjusted Poisson Regression Models

Table 2 shows the unadjusted and adjusted prevalence ratio (APR) of HPV vaccination. Compared with US-born adults, foreign-born adults were approximately 30% less likely to receive the HPV vaccination (APR 0.72; 95% CI, 0.62–0.82).

The initial unadjusted model suggested significant differences in vaccination rates in the homogeneous racial and ethnic groups (NH Black: PR 0.87; 95% CI, 0.77–0.98 and Hispanic: PR 0.84; 95% CI, 0.77–0.92) compared with NH White individuals. However, after controlling for potential confounders, there were no differences in the rates by race/ethnicity. In subsequent models stratified by nativity (Table 2), foreign-born NH Black individuals (APR 0.31; 95% CI, 0.13–0.70) were less likely to be vaccinated against HPV compared with foreign-born NH White individuals, while the US-born NH Asian group (APR 1.27; 95% CI, 1.09–1.48) had a higher prevalence of the vaccination than the US-born NH White group. Additional models comparing US-born vs. foreign-born racial-ethnic subgroups indicated that foreign-born NH Asian (APR 0.60; 95% CI, 0.46–0.77), NH Black (APR 0.27; 95% CI, 0.12–0.61), and Hispanic (APR 0.76; 95% CI, 0.60–0.97) individuals were less commonly vaccinated against HPV compared with their respective US-born counterparts.

## 4. Discussion

The overall and subgroup HPV vaccination rates among adults aged 18 to 26 years were below the Healthy People’s target of 80% HPV vaccination [20]. The overall vaccination rate for male young adults was much lower than that of their female counterparts (37.8% vs. 57.2%). Consistent with a previous study [31], HPV vaccination rates were similar in the non-disaggregated racial-ethnic groups. However, we found substantial disparities in the vaccination rates between foreign-born and US-born population groups. Compared with US-born individuals, foreign-born adults less frequently reported being vaccinated against HPV across all racial/ethnic groups. Specifically, foreign-born NH Black individuals had the lowest prevalence of HPV vaccination and were more than 70% less likely to be vaccinated compared to their US-born-counterparts. Similarly, foreign-born Hispanic had the second lowest vaccination prevalence and were 24% less likely to be vaccinated compared to their US-born counterparts. In contrast, US-born NH Asian young adults had the highest HPV vaccination rate (65.8%), but their foreign-born counterparts were 40% less likely to be vaccinated.

These findings have important implications for HPV cancer prevention in the US. Historically, significant attention has been placed on female HPV-associated diseases, particularly cervical cancer, and its prevention (specifically, screening women and vaccinating young girls). Although men are far less likely to develop high-risk HPV-associated cancers, they serve as the reservoir of the infection while remaining asymptomatic [35,36,37,38]. They can also develop diseases from low-risk HPV (e.g., genital warts) and high-risk HPV (e.g., anal and penile cancers) [36]. Thus, it is critical to develop targeted interventions to increase HPV vaccine uptake among men to reduce the burden of HPV-associated diseases and eventually eliminate the diseases. Furthermore, our results suggest that immigrant adult populations may have higher barriers to vaccination than their US-born counterparts. Additionally, racially and ethnically minoritized immigrant populations may face additional challenges to equitable vaccine access [27]. The results can be attributed to a combination of individual, interpersonal, and structural determinants. Prior studies showed that barriers to HPV vaccination among racial–ethnic minorities include medical distrust, safety concerns, lack of provider recommendations, and inadequate HPV knowledge [25]. On the other hand, among immigrant populations, reported barriers were lack of HPV awareness/knowledge, language barriers, cost, healthcare system navigation issues, and cultural beliefs [39,40,41,42]. Particularly, studies of NH Black immigrants, the group with the lowest vaccination rate in the current study, suggested lack of awareness or provider recommendations, and low perceived susceptibility as major barriers to HPV vaccination uptake [43,44]. For Hispanic populations, commonly reported barriers are lack of knowledge/awareness, sexuality-related parental misconceptions, language barriers, and lack of provider recommendations [45,46].

Immigrant populations are a large portion of the US population. An estimated one in five NH Black individuals in the US are immigrants [47]. The sources of US Black immigrants include Africa, Caribbean countries, and Central and South America. Furthermore, the NH Black immigrant population is one of the fastest-growing immigrant groups in the US with a 475% increase from 800,000 in 1980 to 4.6 million in 2019, which was projected to reach 9.5 million by 2060 [47]. Also, Hispanic individuals are the largest and second-fastest growing immigrant minority population in the US. The rapid growth in the NH Black and Hispanic immigrant populations has critical implications for HPV-associated cancer prevention in the US.

### 4.1. Recommendations

The findings of this study have significant implications for researchers and policymakers. There are profound HPV vaccination inequalities among young adults eligible for catch-up vaccination, affecting racially minoritized foreign-born young adults in the US. The inequalities could slow down the elimination of HPV-related cancers, including cervical cancer, despite the availability of effective and safe vaccination. Effective strategies to reduce racial/ethnic- and nativity-specific barriers are urgently needed to increase HPV vaccine uptake among US immigrant adults to reduce HPV-associated cancer burden. Hence, researchers should partner with institutions and groups that provide services for or interact with the affected subgroups, including colleges, community health centers, consulates, churches, youth group leaders, and relevant social media influencers, to understand the underpinning factors driving the disparities and develop effective feasible collaborative solutions to address barriers to HPV vaccination equity. Additionally, other inherent barriers to HPV vaccination, such as cost, fear of needles, and pain at injection [48], should be considered when designing future intervention studies. Strategies for increasing the vaccine uptake should include tailored HPV prevention and vaccine safety education for eligible male and female populations, free or discounted vaccine resources for uninsured and underinsured individuals eligible for catch-up vaccination, and developing user-friendly (non-injectable) single-dose HPV vaccines.

### 4.2. Strengths and Limitations

The study’s strengths include using a nationally representative sample of young adults in the US. Study limitations include using self-reported surveys that were not validated with medical records and are subject to potential recall bias that could impact the study results. Like other national survey databases, NHIS data collection was affected by the COVID-19 pandemic, which may explain the lower response rate in 2022 compared to 2019. Also, due to the small sample size of foreign-born populations, we could not conduct additional analyses disaggregating the sample by sex and country of birth. We also could not adjust for additional potential confounders, such as length of stay in the US, because of missing observations that could further jeopardize our sample size. Additionally, the outcome of interest was based on the receipt of at least one dose of HPV vaccination, which did not allow for the assessment of the vaccine initiation and completion separately. Despite these limitations, the findings of the study provide important insights that could inform future exploratory and intervention studies.

## 5. Conclusions

In conclusion, despite the availability of safe and effective vaccines for HPV-related cancer prevention, and expansion of recommendation of eligibility age, the findings of this study highlight profound HPV vaccination inequalities among US young adults aged 18–26 years, which were particularly exacerbated among racially and ethnically minoritized immigrant populations. Our results suggest that racially and ethnically minoritized immigrants groups may encounter additional barriers to equitable HPV vaccination access. There is a need to develop effective collaborative catch-up HPV vaccination solutions tailored to reduce vaccination barriers with nativity and race-specific considerations for US young adults who may have missed the HPV vaccination during their adolescent years.

## Figures and Tables

**Table 1:** Weighted sample characteristics and HPV vaccination rates among US young adults aged 18–26 years.

Characteristics	Total N = 4724 (100%)	HPV Vaccination		
		Yes N = 2280 (47.5%)	No N = 2444 (52.5%)	*p*-Value ^a^
Sex				<0.001
Male	2267 (49.8)	869 (37.8)	1398 (62.2)	
Female	2457 (50.2)	1411 (57.2)	1046 (42.8)	
Race and Ethnicity ^b^				0.001
NH Asian	327 (5.6)	168 (51.5)	159 (48.5)	
NH Black	545 (12.9)	233 (43.8)	312 (56.2)	
Hispanic	1127 (22.9)	481 (42.1)	646 (57.9)	
NH Other ^c^	212 (4.6)	108 (48.0)	104 (52.0)	
NH White	2513 (54.0)	1290 (50.3)	1223 (49.7)	
Poverty-Income Ratio				0.022
<100% poverty level	808 (15.4)	372 (46.1)	436 (53.9)	
100–199% poverty level	1018 (20.8)	451 (43.3)	567 (56.7)	
≥200% poverty level	2898 (63.7)	1457 (49.3)	1441 (50.7)	
Health Insurance				<0.001
No	698 (15.3)	203 (27.9)	495 (72.1)	
Yes	4026 (84.7)	2077 (51.1)	1949 (48.9)	
Nativity Status				<0.001
Foreign-Born ^d^	603 (11.9)	204 (31.9)	399 (68.1)	
US-Born	4121 (88.1)	2076 (49.7)	2045 (50.3)	
US Census Region of Residence				0.009
Northeast	676 (16.7)	355 (52.2)	321 (47.8)	
North Central/Midwest	1053 (21.5)	543 (50.2)	510 (49.8)	
South	1784 (38.3)	785 (43.9)	999 (56.1)	
West	1211 (23.5)	597 (47.7)	614 (52.3)	

Abbreviations: HPV, human papillomavirus; NH, non-Hispanic. ^a^ *p*-value from Chi-square test. ^b^ Race/ethnicity groups are mutually exclusive, and Hispanic/Latinos could be of any race. ^c^ Other includes American Indian/Alaska Native, Asian, Native Hawaiian/Pacific Islander, and multiple races. ^d^ Respondents were considered foreign-born if they reported that they were not born in the United States or any of its territories.

**Table 2:** Unadjusted and adjusted prevalence ratios of HPV vaccination among individuals aged 18–26 years by race/ethnicity and nativity status.

Characteristics	Received at Least 1 Dose of HPV Vaccine			
	Unadjusted Model		Adjusted Model ^d^	
	PR (95% CI)	*p*-Value	APR (95% CI)	*p*-Value
Nativity Status ^a^				
Foreign-Born	0.64 (0.56–0.74)	<0.001	0.72 (0.62–0.82)	<0.001
US-Born	reference		reference	
Race and Ethnicity ^b^				
NH Asian	1.02 (0.89–1.18)	0.759	1.01 (0.88–1.16)	0.877
NH Black	0.87 (0.77–0.98)	0.023	0.91 (0.81–1.03)	0.136
Hispanic	0.84 (0.77–0.92)	<0.001	0.93 (0.85–1.02)	0.113
NH Other ^c^	0.95 (0.80–1.14)	0.614	1.00 (0.84–1.19)	0.976
NH White	reference		reference	
Foreign-Born ^a^ by Race and Ethnicity ^b^				
NH Asian	0.80 (0.56–1.14)	0.215	0.86 (0.61–1.21)	0.382
NH Black	0.25 (0.11–0.58)	0.001	0.31 (0.13–0.70)	0.005
Hispanic	0.61 (0.43–0.88)	0.007	0.89 (0.62–1.26)	0.495
NH Other ^c^	0.40 (0.15–1.02)	0.055	0.52 (0.20–1.35)	0.179
NH White	reference		reference	
US-Born ^a^ by Race and Ethnicity ^b^				
NH Asian	1.31 (1.12–1.52)	0.001	1.27 (1.09–1.48)	0.003
NH Black	0.93 (0.82–1.04)	0.203	0.96 (0.85–1.08)	0.471
Hispanic	0.93 (0.84–1.02)	0.132	0.97 (0.88–1.07)	0.529
NH Other ^c^	1.01 (0.84–1.21)	0.936	1.04 (0.88–1.24)	0.635
NH White	reference		reference	
Foreign-born vs. US-Born ^a^ by Racial-Ethnic ^b^ groups (Reference = US-Born)				
Foreign-born vs. US-Born: NH Asian	0.58 (0.45–0.75)	<0.001	0.60 (0.46–0.77)	<0.001
Foreign-born vs. US-Born: NH Black	0.26 (0.11–0.57)	0.001	0.27 (0.12–0.61)	0.002
Foreign-born vs. US-Born: Hispanic	0.63 (0.49–0.80)	<0.001	0.76 (0.60–0.97)	0.025
Foreign-born vs. US-Born: NH Other ^c^	0.37 (0.14–0.97)	0.043	0.46 (0.17–1.21)	0.116
Foreign-born vs. US-Born: NH White	0.95 (0.72–1.25)	0.715	0.91 (0.69–1.20)	0.504

Abbreviations: HPV, human papillomavirus; NH, non-Hispanic. ^a^ Respondents were considered foreign-born if they reported that they were not born in the United States or any of its territories. ^b^ Race/ethnicity groups are mutually exclusive, and Hispanic/Latinos could be of any race. ^c^ Other includes American Indian/Alaska Native, Asian, Native Hawaiian/Pacific Islander, and multiple races. ^d^ Models adjusted for sex, health insurance, poverty–income ratio, and US census region of residence.

## Data Availability

Data Availability Statements are available at https://healthsurveys.ipums.org/, accessed on 19 May 2024..
